# Batch adjustment by reference alignment (BARA): Improved prediction performance in biological test sets with batch effects

**DOI:** 10.1371/journal.pone.0212669

**Published:** 2019-02-22

**Authors:** Robin Gradin, Malin Lindstedt, Henrik Johansson

**Affiliations:** 1 SenzaGen AB, Lund, Sweden; 2 Department of Immunotechnology, Lund University, Lund, Sweden; Arizona State University, UNITED STATES

## Abstract

Many biological data acquisition platforms suffer from inadvertent inclusion of biologically irrelevant variance in analyzed data, collectively termed batch effects. Batch effects can lead to difficulties in downstream analysis by lowering the power to detect biologically interesting differences and can in certain instances lead to false discoveries. They are especially troublesome in predictive modelling where samples in training sets and test sets are often completely correlated with batches. In this article, we present BARA, a normalization method for adjusting batch effects in predictive modelling. BARA utilizes a few reference samples to adjust for batch effects in a compressed data space spanned by the training set. We evaluate BARA using a collection of publicly available datasets and three different prediction models, and compare its performance to already existing methods developed for similar purposes. The results show that data normalized with BARA generates high and consistent prediction performances. Further, they suggest that BARA produces reliable performances independent of the examined classifiers. We therefore conclude that BARA has great potential to facilitate the development of predictive assays where test sets and training sets are correlated with batch.

## Introduction

Data acquisition techniques designed to quantify biological signals from gene- or protein expression are often associated with batch effects. The problem with batch effects is that it leads to the inclusion of biologically irrelevant variance in the obtained data, which can lower the power of subsequent analyses or lead to false discoveries [1–4]. The variance may be due to a variety of different experimental parameters, including analysis date, sample processing or reagent quality [5]. Further, batch effects are not exclusive to high throughput acquisition methods but are also observed in data from lower throughput methods such as qPCR or NanoString nCounter technologies [6, 7]. The high incidence of batch effects in multiple biological platforms is a contributing factor to the relatively small number of diagnostic and prognostic biomarker signatures that have been implemented in clinical settings [6, 8].

Some actions have been shown to reduce the impact of batch effects. One such action is to carefully design experiments to minimize the correlation between possible sources of technical variance and known biological factors. However, this action is not possible for all types of experiments. For predictive modelling, for example, the correlation between biological factors and batches cannot be eliminated. This is due to the inherent nature of these experiments, where fixed training sets are often used to infer parameter values used to predict subsequently acquired test sets. This leads to total confoundment between batches and samples in the test sets, which can result in poor predictive performances on test sets [9]. Another option to reduce the impact of batch effects is to apply analytical methods on already obtained data. Many such methods have been designed, but most require prior knowledge of the biological factors of interest and low confoundment between batches and the biological groups [10–12]. Examples of such methods are ComBat and surrogate variable analysis (SVA). ComBat is a supervised batch correction method that requires that the sources of batch effects are known. It is a location and scale method that uses the empirical Bayes method to moderate the batch effect estimates, making it better equipped to handle smaller datasets [10]. In contrast to ComBat and other supervised batch effect adjustment methods, SVA does not require that the sources of batch effects are known. Instead, the biological sources of interest should be known and specified in the model. The initial step of SVA estimates and removes the variance associated with the known biological information. Latent structures are then identified in the residual matrix, which can either be removed to generate a cleaned dataset or be incorporated in subsequent significant analyses. Identified latent structures can contain information linked to batch effects, but they can also contain other sources of expression heterogeneity, such as biological factors not included in the initial modeling [11]. Both SVA and ComBat were originally developed for datasets in discovery studies, where biological sources of interest and possible sources of batch effects are known. Because of this, they and other methods developed for similar purposes are not directly applicable to datasets generated in predictive settings. However, by making certain assumptions, the algorithms can be modified to be used in predictive modelling. For ComBat, one must assume that the composition of test sets is similar to that in the training set. But this assumption can be violated when, for example, the size of the test set decreases, as shown in [13]. For SVA, one can assume that latent structures identified in the training set can also be identified in test sets. This assumption was used to develop the frozen SVA algorithm [14]. However, this assumption is not valid if latent structures associated with batch effects are different in the training set and test sets. This can lead to poor predictive performances as shown in [13]. In general, for a normalization method to be applicable in a wide range of prediction problems, it should allow for training sets and test sets to be correlated with batch. Further, the training set should not be altered when normalizing with different test sets. Finally, it should ideally allow test sets to be acquired without the need to include a large amount of reference samples.

In this paper, we introduce *Batch Adjustment by Reference Alignment* (BARA) to adjust for batch effects in predictive modelling. The method has the advantage that only a few reference samples are necessary to perform batch corrections. Also, rather than attempting to clean the data by removing batch effects from both training set and test sets, BARA aims to transform the test set to make it more similar to the training set. BARA performs the adjustment in a compressed data space spanned by the training set, thereby alleviating the number of necessary batch estimates that needs to be performed. We test the BARA method on a collection of 25 publicly available datasets and show that BARA consistently aids the classifier to achieve high prediction performances. We further show that the performance of BARA is better or comparable to the performance of existing methods on the examined datasets. By reducing the negative impact of batch effects, the prediction performances observed with BARA can facilitate the development of predictive assays.

## Materials and methods

The R software environment was used to perform the analyses presented in this paper [15]. Figures were created with the R-package ggplot2 [16]. In addition, the following R-packages were used; reshape2, dplyr, stringr, data.table, magrittr, foreach, doParallel, e1071, randomForest, class and bapred [17–27]. The scripts used to generate the results, including the BARA algorithm, are available at: https://github.com/gradinetal2018/BARA.

### Cross-study datasets

25 datasets compiled by Hornung et. al. [13] were downloaded from ArrayExpress [28], see Table 1. The gene expression levels of all datasets were quantified with Affymetrix GeneChip Human Genome U133 Plus 2.0. The raw data files (CEL files) were normalized using single channel array normalization [29]. For each dataset, duplicated samples were removed, and only samples with existing annotations of gender were retained. All samples were annotated by *gender/sex*.

**Table 1:** Datasets used in the cross-study analysis.

Accession Number	Sample Size	Reference
E-GEOD-19722	46	[30]
E-GEOD-28654	112	[31]
E-GEOD-29623	65	[32]
E-GEOD-39084	70	[33]
E-GEOD-45216	31	[34]
E-GEOD-45670	38	[35]
E-GEOD-46474	40	[36]
E-GEOD-48278	57	[37]
E-GEOD-48350	83	[38, 39]
E-GEOD-48780	49	[40]
E-GEOD-49243	73	[41, 42]
E-GEOD-50774	21	[43]
E-GEOD-53224	53	[44]
E-GEOD-53890	41	[45]
E-GEOD-54543	30	[46]
E-GEOD-54837	226	[47]
E-GEOD-58697	124	[48]
E-GEOD-59312	79	[49]
E-GEOD-60028	24	[50]
E-GEOD-61804	325	[51]
E-GEOD-63626	63	[52]
E-GEOD-64415	209	[53]
E-GEOD-64857	81	[54]
E-GEOD-67851	31	[55]
E-GEOD-68720	97	[56]

The table describes each dataset’s accession number and the number of samples extracted from it.

### Cross-study prediction evaluation

Cross-study prediction performances were used to evaluate the performance of BARA and to compare it to existing normalization methods. The normalization methods included in this analysis were; BARA, ComBat [10], FABatch [21], fSVA exact [14], mean centering, ratio A, reference centering, reference ratio A and standardization. The reference centering method subtracts the reference samples’ mean expression of each gene from all samples in the training set and the test set respectively. Similarly, the reference ratio A method scales each gene and sample in the training set and the test set by their respective reference samples mean expression. To examine the predictive performance on normalized data, each of the 25 datasets was iteratively used as a temporary training set. First, 3 samples from the same biological group were randomly selected as reference samples in the training set. Next, using all samples in the training set, the 500 most significant differentially expressed genes, comparing males to females, were identified using limma [57, 58]. Because the normalization methods had all been adjusted to be used in predictive modelling, as implemented in the bapred package [21] or through implementations in R, each method was first applied to the training set. Next, the transformed training set was used to define the prediction models. Three different prediction models were examined; k-nearest neighbors (kNN), random forest, and support vector machines (SVM). The prediction models were tuned using repeated cross-validation on the training set, with 3 repeats and 10 folds. The parameters resulting in the highest mean prediction performance, evaluated using Mathews Correlation Coefficient (MCC), were selected to establish the final prediction model. To allow for variation among the samples acting as reference sample, the test set normalization and prediction procedure was repeated 10 times for each test set, using a different selection of reference samples in each iteration. More specifically, when classifying the samples in each test set, 3 samples from the same group as the reference samples in the training set were randomly selected from the test set. The normalization methods that did not rely on reference samples used all samples in the test set, including the 3 reference samples, while the reference-based normalization methods only used the reference samples to normalize the data. Because information about the group of the reference samples could be considered being leaked during the normalization procedure, the reference samples were removed from the test sets before the predictions were made. The final prediction performance for each test set was calculated as the median MCC from the 10 iterations. To obtain an overall prediction performance for each training set, the MCCs of the 24 test sets were averaged. A summarized prediction score for each normalization and prediction model was calculated as the mean MCC from all the training sets.

### Assessment of BARA’s dependence on the number of reference samples

To assess the performance of the BARA algorithm as the number of reference samples was varied, the cross-study prediction approach described above was repeated. The performance estimation was repeated 6 times, where the number of utilized reference samples was varied from 1 sample to 6 samples. The predictive performances were summarized as described above.

### The BARA algorithm

The BARA algorithm was created specifically for predictive modelling, where a fixed training set is used to classify test samples possibly affected by batch effects. The training set is used to identify a set of directions that captures the largest part of the variance in the data, using singular value decomposition (SVD). This step allows the data to be compressed into a lower dimensional space, which reduces the number of necessary batch estimates, and simultaneously decreases the complexity of the data.

The training dataset, X, contains m samples in rows and n variables in columns. Each variable in X is centered by its mean value, and the matrix is decomposed using SVD to identify the directions where the batch adjustment is performed.

$$X_{0}=\sum_{i=1}^{m}\sum_{j=1}^{n}X_{ij}-{\bar{x}}_{j}$$ 1

$${USV}^{T}=SVD(x)$$ 2

where $\bar{x}$ represents a 1*n dimensional vector containing the column means of X, U is the left singular vectors, S the singular values, and V the right singular vectors. The number of dimensions retained, *k*, is an adjustable parameter that can be set by using a predetermined value, estimated with for example cross-validation, or determined by setting an acceptable loss of variance in the training set, for example 10%. The centered training data is then multiplied by the first *k* columns of the right singular vector to obtain a transformed training set.

$$X^{\prime }=X_{0}*V_{,1:k}$$ 3

The test dataset, Z, contains p samples in rows and n variables. The variables are first adjusted by subtracting the mean values of the training data, and is then projected onto the identified directions.

$$Z_{0}=\sum_{i=1}^{p}\sum_{j=1}^{n}Z_{ij}-{\bar{x}}_{j}$$ 4

$$Z^{\prime }=Z_{0}*V_{,1:k}$$ 5

A batch adjustment factor, a_j_, is estimated for all retained dimensions, using the reference samples present in both the training set and the test set. For example, the adjustment factor of dimension *j* is estimated by comparing the mean value of the reference samples in the training set for dimension *j*, to the mean value of the reference samples in the test set for dimension *j*.

$$a_{j}={\bar{z}}_{ref,j}^{\prime }-{\bar{x}}_{ref,j}^{\prime }$$ 6

Where ${\bar{z}}_{\mathrm{ref}}$ and ${\bar{x}}_{\mathrm{ref}}$ are 1*k dimensional vectors containing the variable means for the reference samples in the transformed test set and the transformed training set respectively. The transformed test data is then adjusted by the adjustment factors and both datasets are reconstructed to the original data space.

$$Z^{\prime }=\sum_{i=1}^{p}\sum_{j=1}^{k}Z_{ij}^{\prime }-a_{j}$$ 7

$$X^{k}=X^{\prime }*V_{,1:k}^{T}$$ 8

$$Z^{k}=Z^{\prime }*V_{,1:k}^{T}$$ 9

To achieve a level of expression to what was originally observed, the mean values estimated from the training set are finally added to the reconstructed data.

$$X^{k}=\sum_{i=1}^{m}\sum_{j=1}^{n}X_{ij}^{k}+{\bar{x}}_{j}$$ 10

$$Z^{k}=\sum_{i=1}^{p}\sum_{j=1}^{n}Z_{ij}^{k}+{\bar{x}}_{j}$$ 11

The predictions are then performed as normal, where the model is built from the compressed training dataset, X^k^, and the predictions are made on the adjusted test set, Z^k^.

### Normalization methods and parameter settings

The following methods were used through their implementations in the R-package bapred; ComBat, FAbatch, fSVA exact, mean centering, ratio A and standardization. For FAbatch, the default values of the parameters were used, i.e. the number of factors to estimate for each batch was left unspecified, the preliminary probabilities were estimated using leave-one-out cross-validation, maximum number of iterations were 100, and the maximum number of factors were 12. For fSVA exact, the algorithm parameter was changed to correspond to the exact algorithm instead of the fast, while the default values were used for the remaining parameters. For the other methods implemented in the bapred package, no additional parameter values could be specified.

The two reference-based methods, reference mean centering and reference ratio A, where implemented in R. Reference mean centering subtracted each batch’s genes by the mean expression of its reference samples, and reference ratio A scaled each batch’s genes expression by the mean expression of the reference samples.

BARA was implemented by specifying the loss parameter as a criterion for selecting the number of dimensions to retain. The loss parameter was set to 10%. Thus, at most 10% of the variance in the training data was lost in the normalization.

### Prediction models

Three types of prediction models were used to assess the performance of the normalization methods in the cross-study analysis. The prediction models were; random forest, kNN and SVM with linear kernel. The prediction models were implemented using the R-packages randomForest, class and e1071 [18, 22, 26]. The prediction models were selected to include both linear and non-linear classifiers. For every training set, the prediction models were tuned to maximize the MCC using repeated cross-validation with 3 repeats and 10 folds. For the respective prediction model, the following parameters and parameter values were tuned:

- kNN
  ○ Number of nearest neighbors: 1, 2, 3, 4, 5, 6, 7, 8, 9
- Random Forest
  ○ Mtry: 5, 7, 9, 10, 11, 13, 15, 17
  ○ Ntree: 500, 1000, 1500, 2000, 2500, 3000
- SVM
  ○ Cost: 0.001, 0.005, 0.01, 0.05, 0.1, 0.5, 1, 5, 10, 50

## Results

**Fig 1:**
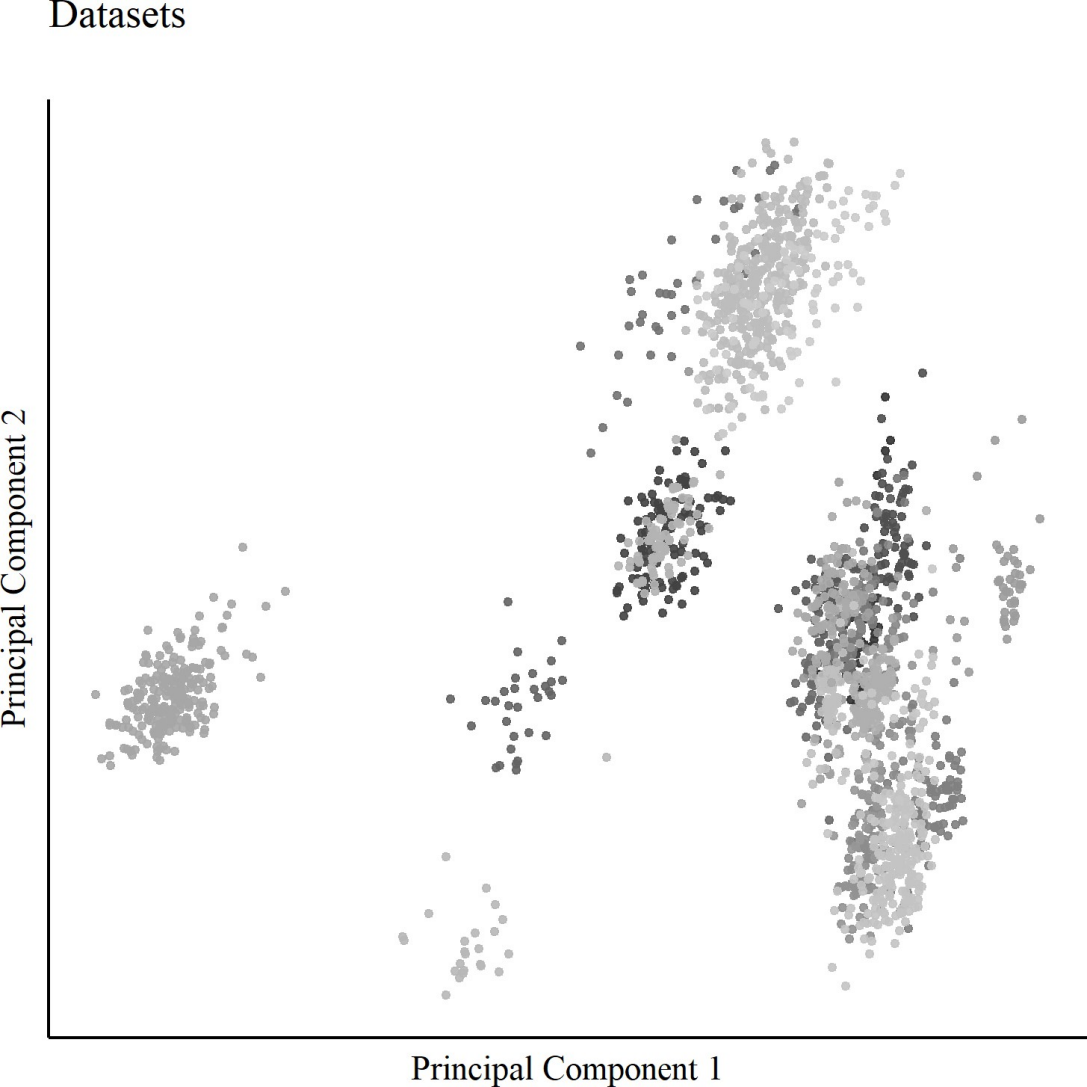
PCA plot of the datasets. The figure shows the first two principal components after merging the acquired datasets. The samples were colored by the datasets from where they originated.

To assess the performance of BARA and to compare it to existing normalization methods, cross-study predictions were examined. Because the acquired datasets originated from separate studies, the biological annotation used for classification was sex/gender. Fig 1 shows a PCA plot of the 25 datasets, which shows clear signs of batch effects.

**Fig 2:**
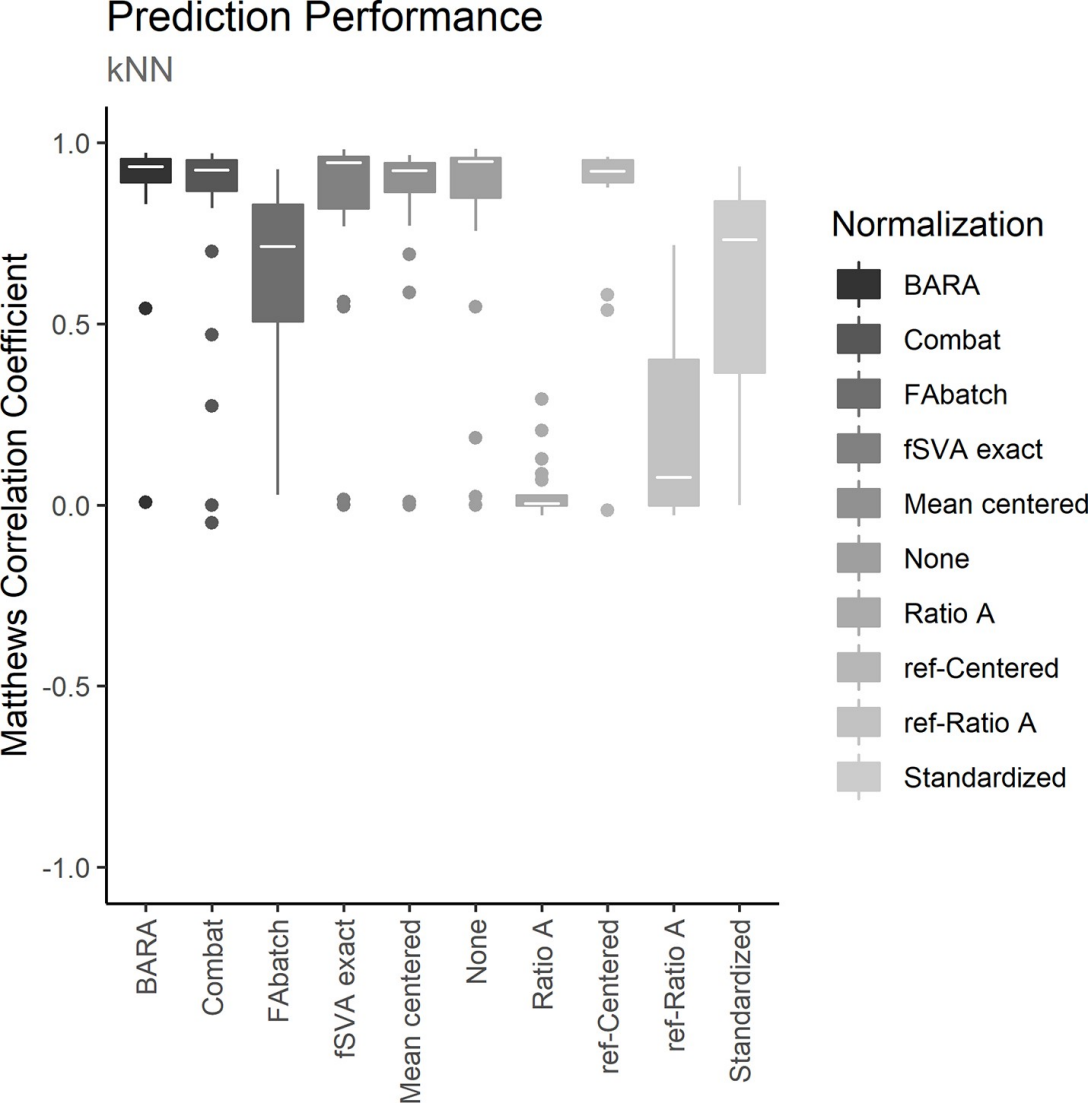
Prediction performance, kNN. The plot shows the predictive performances for the different methods when normalized data were classified with kNN models. The boxes represent the 25 MCCs obtained in the iterative exercise where each dataset was used as training set to classify the remaining datasets.

**Fig 3:**
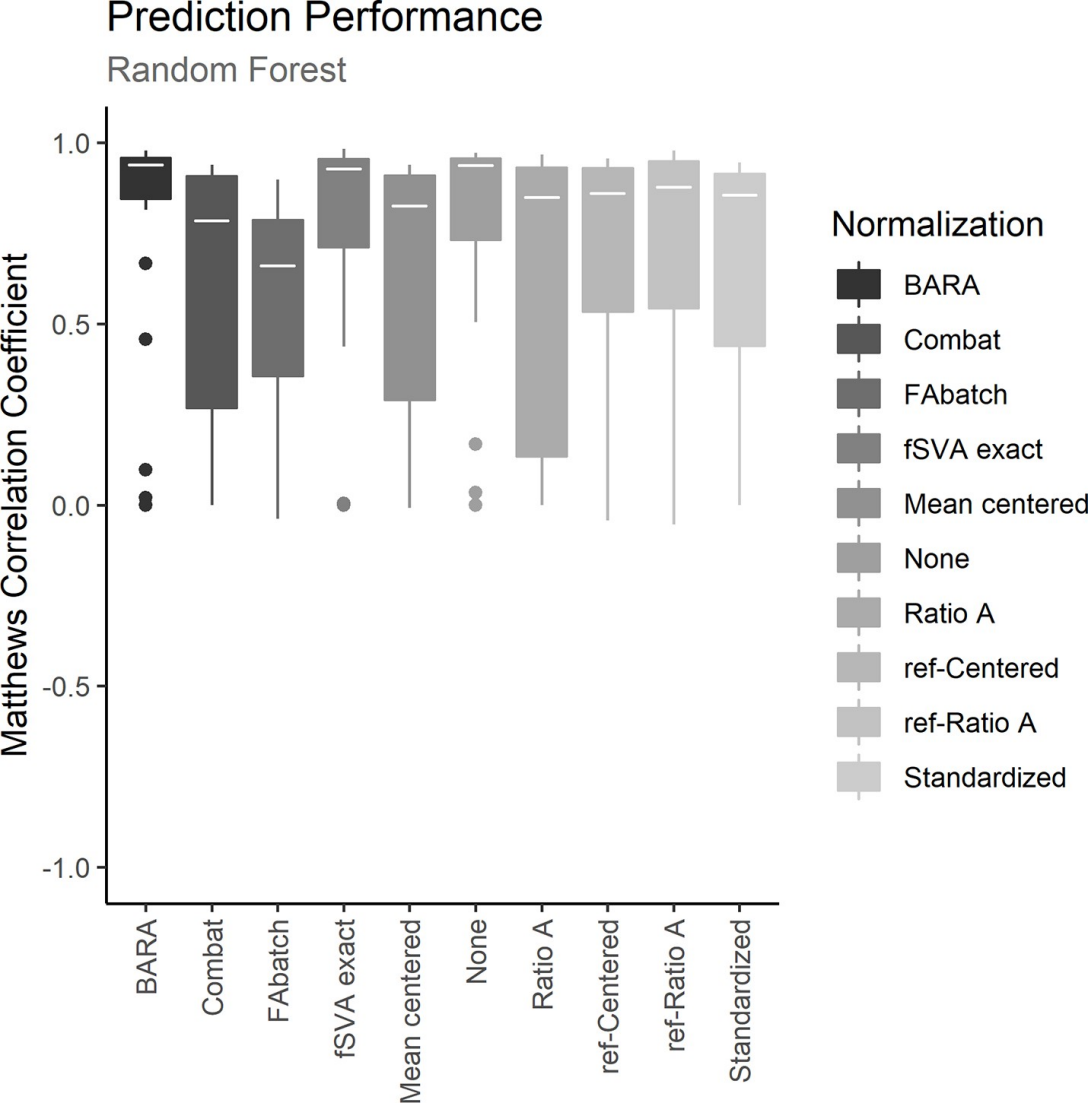
Prediction performance, random forest. The plot shows the predictive performances for the different methods when normalized data were classified with random forest models. The boxes represent the 25 MCCs obtained in the iterative exercise where each dataset was used as training set to classify the remaining datasets.

**Fig 4:**
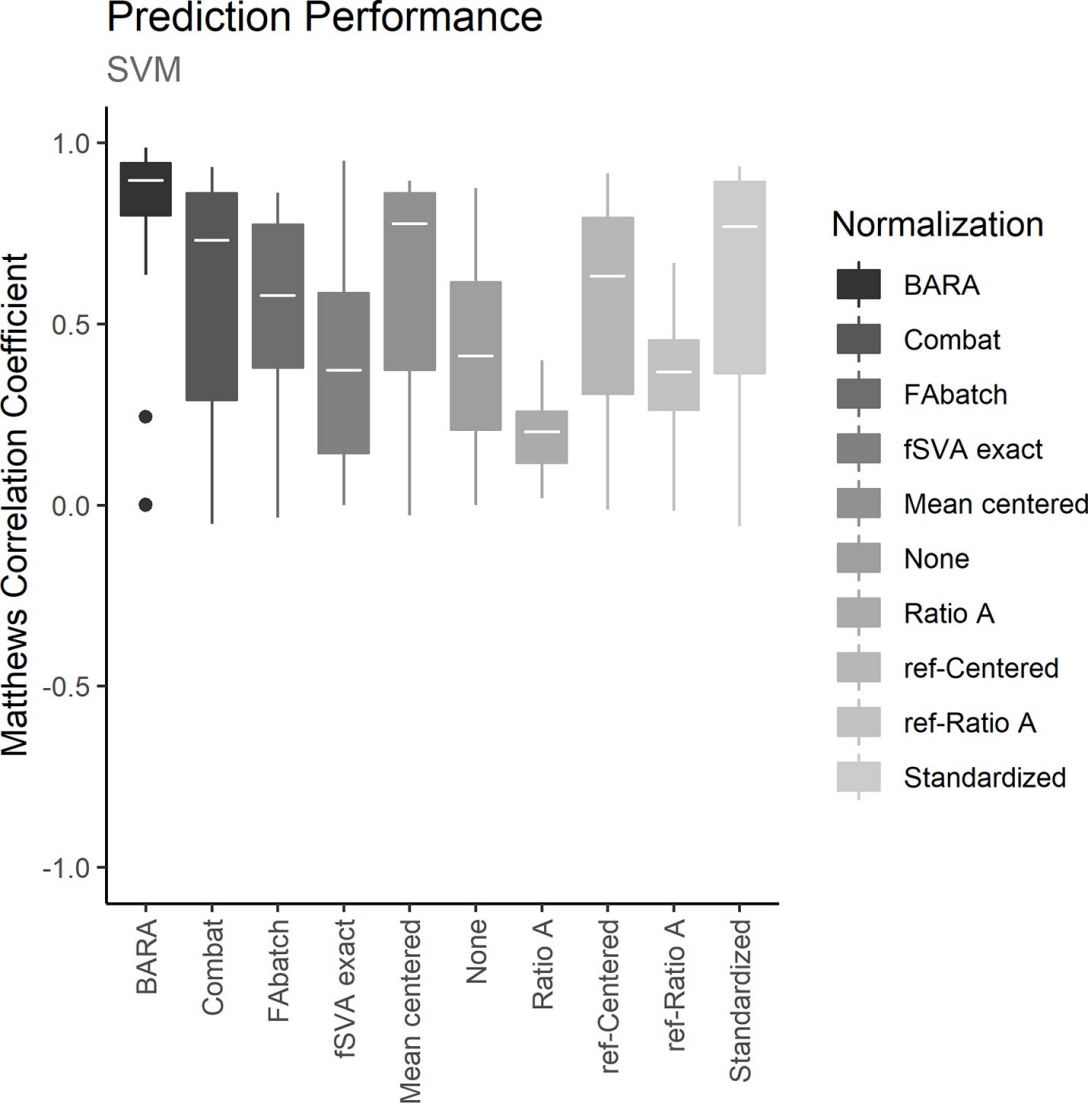
Prediction performance, SVM. The plot shows the predictive performances for the different methods when normalized data were classified with SVMs. The boxes represent the 25 MCCs obtained in the iterative exercise where each dataset was used as training set to classify the remaining datasets.

To assess the different normalization methods, each dataset was iteratively used as training set to define a prediction model. Batch effects between the training sets and the test sets were adjusted with the examined normalization methods. Normalized test sets were classified with the trained prediction models and MCCs were calculated to estimate the prediction performances. The resulting MCCs for the normalized data and for the unnormalized data on the 3 different prediction models are shown in Figs 2–4. Further, the mean performance for each normalization method and prediction model can be seen in Table 2. Figs 2–4 show that data normalized with BARA seems to generate consistent performances independent of the examined prediction model. Further, the estimated MCCs are high with low variance. In fact, considering the calculated performance scores in Table 2, BARA achieves the highest mean MCC compared to the other examined normalization methods. Lastly, BARA also shows an improved prediction performance compared to the unnormalized data.

**Table 2:** Prediction performances.

Normalization Method	kNN	Random Forest	SVM
BARA	**0.88 ± 0.20**	**0.80 ± 0.31**	**0.78 ± 0.28**
ComBat	0.80 ± 0.30	0.60 ± 0.37	0.57 ± 0.36
FAbatch	0.65 ± 0.24	0.55 ± 0.30	0.54 ± 0.27
fSVA Exact	0.82 ± 0.27	0.74 ± 0.35	0.39 ± 0.29
Mean Centered	0.82 ± 0.26	0.63 ± 0.34	0.60 ± 0.31
None	0.81 ± 0.30	0.76 ± 0.34	0.43 ± 0.29
Ratio A	0.04 ± 0.07	0.60 ± 0.39	0.20 ± 0.10
Reference Centered	0.86 ± 0.21	0.70 ± 0.32	0.51 ± 0.31
Reference Ratio A	0.20 ± 0.26	0.71 ± 0.34	0.37 ± 0.16
Standardized	0.60 ± 0.30	0.66 ± 0.36	0.59 ± 0.35

Prediction performances for each normalization and prediction model. Each performance is given as the mean MCC ± the standard deviation.

**Fig 5:**
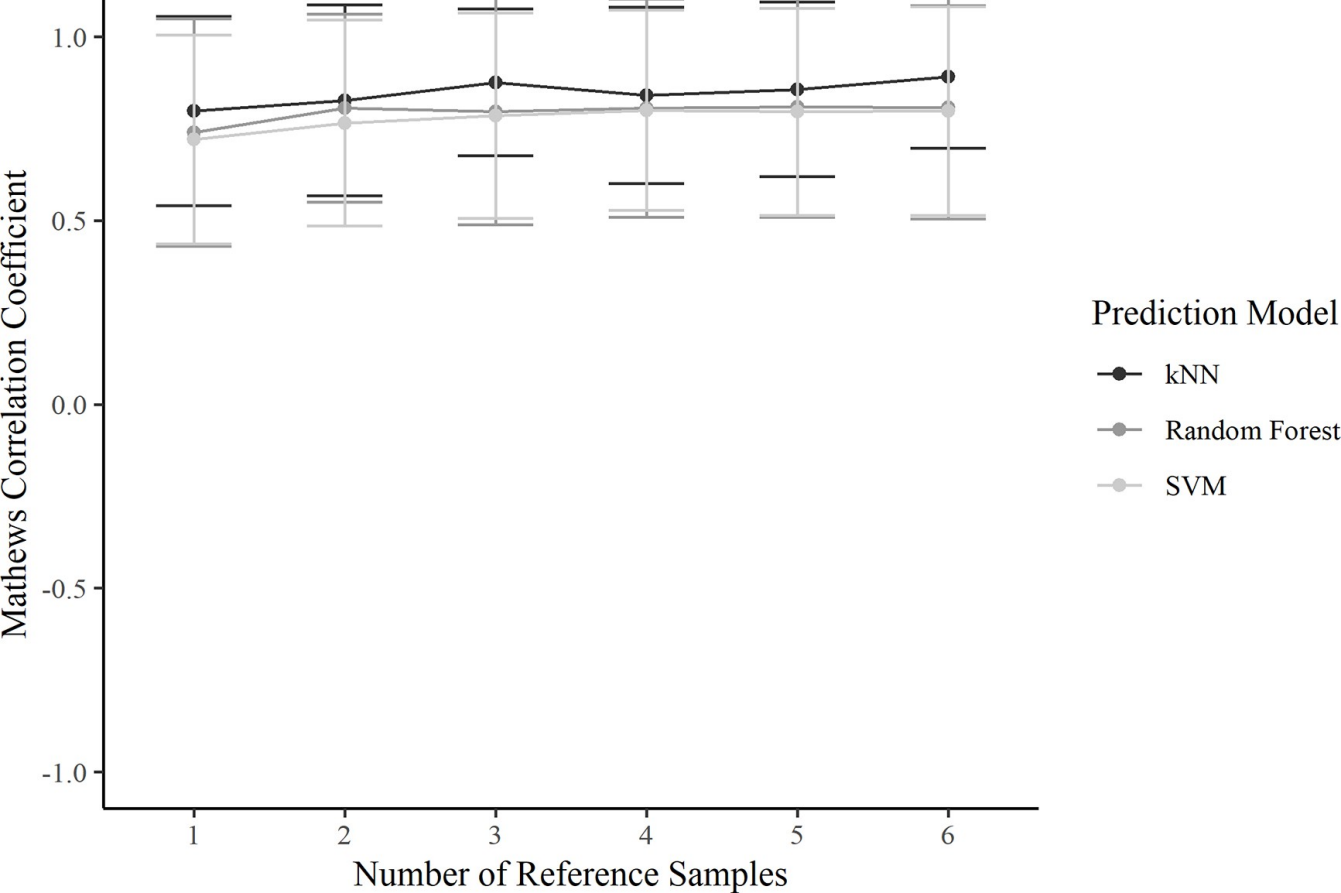
Performance of BARA as the number of reference samples is varied. The points represent the mean MCCs as each of the 25 datasets was iteratively used as training set to classify the remaining samples. The bars represent the standard deviation of the MCC.

Because all information used to adjust for batch effects in the BARA method is estimated from the reference samples, the performance of the method as the number of reference samples was varied was examined. The number of reference samples was varied from 1 sample to 6 samples, and the cross-study prediction approach described above was repeated. Again, the performances were summarized by calculating the mean MCC for each run. The performance for each repeat can be seen in Fig 5. The plot suggests that the performance of BARA in the examined datasets is robust to the number of reference samples utilized. However, it is also evident that the run with a single reference sample achieve the lowest performance metric.

## Discussion

Batch effects are a widespread problem that exist in most biological data acquisition platforms and hinder the development and implementation of promising biomarker signatures [1–5]. Batch effects are especially difficult to account for in predictive modelling where the biological factors in the test set are completely confounded with batch. In this paper, we have introduced BARA, a normalization method for the adjustment of batch effects in predictive modelling, and compared it to already existing methods. The aim of normalization methods applied in settings of predictive modelling is to make prediction models and inferred information transferable to test sets affected by batch effects. Common strategies suggest using reference samples to estimate the perturbation induced by batch effects [9]. However, including multiple additional samples in each batch of test samples can be both time consuming and add analytical costs. Therefore, normalization methods should ideally require none or few reference samples.

Only a few reference samples are required by BARA; three were used in the comparison described above and BARA was also found to achieve robust performance metrics when only a single reference sample was used. However, the optimal number of reference samples could generally be assumed to be based on a trade-off between costs and accuracy. Because the reference samples are used to estimate mean adjustments for every batch in a compressed data space, additional reference samples should provide more certain estimates of the mean values. This was also observed when the number of reference samples used by BARA was varied, see Fig 5. Because mean values are estimated, it is also expected that the ideal number of reference samples will not be the same for different data acquisition techniques and datasets but will depend on, for example, the variation between replicates. Further, even though random assignment of reference samples does not represent an ideal selection strategy, the performance of BARA could be considered stable as indicated by the low variance in the performance metrics, see Figs 2–4. In an ideal scenario, the reference samples should represent a standardized sample where the major difference compared to other reference samples are batch effects. This could lead to better estimates with a lower number of required reference samples.

BARA estimates the batch adjustments in a compressed data space spanned by the training set. The compression is calculated with SVD and is thus a linear combination of the original variables along the directions of maximum variance. SVD was chosen because we hypothesized that it would be suitable for many datasets associated with predictive modelling where biomarker signatures have been identified to optimize prediction performances, which suggests that low-dimensional representations of the data exist that captures large fractions of the important variance. Further, SVD is a well-known operation that offers a convenient way of compressing data, performing batch adjustment and reconstructing the original variables. Because SVD is used in BARA, multiple levels of compression can be selected for a dataset depending on the number of directions that are retained. In the cross-study analyses described above, a maximum of 10% of the variance in the training set was considered an acceptable loss, and the smallest number of dimensions satisfying this condition was selected in the normalization steps. However, other approaches for selecting an optimal number of dimensions to retain in a specific dataset could be pursued. For example, cross-validation performances could be compared as the number of dimensions retained are varied, or an external validation set affected by batch effects could aid the selection by better mimicking an actual prediction scenario.

The ability of BARA to restore the predictive performances in datasets suffering from batch effects was assessed by cross-study predictions, and the obtained performances were compared to those obtained using existing normalization methods or no normalization. Due to the difficulty in procuring public datasets containing training sets suitable for predictive modelling where external test sets affected by batch effects exist, a collection of datasets previously compiled [13] were used, where sex/gender was used as classification label. Successively, each of the 25 datasets in the collection were designated as a training set. From the training set, the 500 most significant genes after comparing the gene expression of females to males were identified, and prediction models were defined using kNN, random forest and SVM. Batch effects between the training sets and the test sets were removed with the examined normalization methods, and the samples in the test set were classified. Figs 2–4 shows the predictive performances after applying the examined normalization methods. The figures show that BARA produces high and consistent performances for the examined datasets independent of prediction model. The results further suggest, that BARA outperforms the other examined normalization methods on the examined datasets, by reaching the highest mean MCC values and consistently show low variation in performance. Further, the performance is also improved compared to using no normalization, which indicates that the BARA algorithm mitigates some of the negative effects caused by batch effects in the studied datasets. It is also worth noting that genes associated with sex/gender are strong predictors, and the performance of the unnormalized data was often higher than those obtained by some of the normalization methods. In fact, BARA was the only normalization method that consistently resulted in improved mean prediction performance compared to the unnormalized data in all three prediction models.

In conclusion, we have introduced a novel method to adjust for batch effects in predictive modelling and compared it to already existing methods. We show that BARA improves the prediction performances in the examined datasets compared to applying no normalization. Further, the BARA-normalized datasets achieved higher or comparable prediction performances compared to datasets normalized with the other examined methods. These results suggest that BARA can be considered a useful tool to reduce the negative impact of batch effect in predictive modelling.

## References

[1] Lambert CG, Black LJ. Learning from our GWAS mistakes: from experimental design to scientific method. Biostatistics (Oxford, England). 2012;13(2):195–203. 10.1093/biostatistics/kxr055 PMC3297828. 22285994 PMC3297828

[2] Leek JT, Scharpf RB, Bravo HC, Simcha D, Langmead B, Johnson WE, et al Tackling the widespread and critical impact of batch effects in high-throughput data. Nature Reviews Genetics. 2010;11:733 10.1038/nrg2825 https://www.nature.com/articles/nrg2825#supplementary-information. 20838408 PMC3880143

[3] McLerran D, Grizzle WE, Feng Z, Thompson IM, Bigbee WL, Cazares LH, et al SELDI-TOF MS Whole Serum Proteomic Profiling with IMAC Surface Does Not Reliably Detect Prostate Cancer. Clinical chemistry. 2008;54(1):53–60. 10.1373/clinchem.2007.091496 PMC4332515. 18024530 PMC4332515

[4] Tung P-Y, Blischak JD, Hsiao CJ, Knowles DA, Burnett JE, Pritchard JK, et al Batch effects and the effective design of single-cell gene expression studies. Scientific reports. 2017;7:39921 10.1038/srep39921 https://www.nature.com/articles/srep39921#supplementary-information. 28045081 PMC5206706

[5] Scherer A. Batch effects and noise in microarray experiments: sources and solutions: John Wiley & Sons; 2009.

[6] Goh WWB, Wang W, Wong L. Why Batch Effects Matter in Omics Data, and How to Avoid Them. Trends in Biotechnology. 35(6):498–507. 10.1016/j.tibtech.2017.02.012 28351613

[7] Talhouk A, Kommoss S, Mackenzie R, Cheung M, Leung S, Chiu DS, et al Single-Patient Molecular Testing with NanoString nCounter Data Using a Reference-Based Strategy for Batch Effect Correction. PLOS ONE. 2016;11(4):e0153844 10.1371/journal.pone.0153844 27096160 PMC4838303

[8] Diamandis EP. Cancer Biomarkers: Can We Turn Recent Failures into Success? JNCI Journal of the National Cancer Institute. 2010;102(19):1462–7. 10.1093/jnci/djq306 PMC2950166. 20705936 PMC2950166

[9] Luo J, Schumacher M, Scherer A, Sanoudou D, Megherbi D, Davison T, et al A comparison of batch effect removal methods for enhancement of prediction performance using MAQC-II microarray gene expression data. The Pharmacogenomics Journal. 2010;10(4):278–91. 10.1038/tpj.2010.57 PMC2920074. 20676067 PMC2920074

[10] Johnson WE, Li C, Rabinovic A. Adjusting batch effects in microarray expression data using empirical Bayes methods. Biostatistics. 2007;8(1):118–27. 10.1093/biostatistics/kxj037 16632515

[11] Leek JT, Storey JD. Capturing Heterogeneity in Gene Expression Studies by Surrogate Variable Analysis. PLOS Genetics. 2007;3(9):e161 10.1371/journal.pgen.0030161 17907809 PMC1994707

[12] Oytam Y, Sobhanmanesh F, Duesing K, Bowden JC, Osmond-McLeod M, Ross J. Risk-conscious correction of batch effects: maximising information extraction from high-throughput genomic datasets. BMC Bioinformatics. 2016;17(1):332 10.1186/s12859-016-1212-5 27585881 PMC5009651

[13] Hornung R, Causeur D, Bernau C, Boulesteix AL. Improving cross-study prediction through addon batch effect adjustment or addon normalization. Bioinformatics (Oxford, England). 2017;33(3):397–404. Epub 2016/11/01. 10.1093/bioinformatics/btw650 . 27797760

[14] Parker HS, Corrada Bravo H, Leek JT. Removing batch effects for prediction problems with frozen surrogate variable analysis. PeerJ. 2014;2:e561 Epub 2014/10/22. 10.7717/peerj.561 25332844 PMC4179553

[15] Team RC. R: A Language and Environment for Statistical Computing. 2018.

[16] Wickham H. ggplot2: Elegant Graphics for Data Analysis: Springer-Verlag New York; 2009.

[17] Corporation M, Weston S. doParallel: Foreach Parallel Adaptor for the 'parallel' Package. 2017.

[18] David M, Evgenia D, Kurt H, Andreas W, Friedrich L. e1071: Misc Functions of the Department of Statistics, Probability Theory Group (Formerly: E1071), TU Wien. 2018.

[19] Hadley W. stringr: Simple, Consistent Wrappers for Common String Operations. 2018.

[20] Hadley W, Romain F, Lionel H, Kirill M. dplyr: A Grammar of Data Manipulation. 2017.

[21] Hornung R, Boulesteix A-L, Causeur D. Combining location-and-scale batch effect adjustment with data cleaning by latent factor adjustment. BMC Bioinformatics. 2016;17(1):27 10.1186/s12859-015-0870-z 26753519 PMC4710051

[22] Liaw A, Wiener M. Classification and Regression by randomForest. R news. 2002;2(3):18–22.

[23] Matt D, Arun S. data.table: Extension of `data.frame. 2017.

[24] Microsoft, Steve W. foreach: Provides Foreach Looping Construct for R. 2017.

[25] Stefan MB, Hadley W. magrittr: A Forward-Pipe Operator for R. 2014.

[26] Venables WN, Ripley BD. Modern Applied Statistics with S. Fourth ed: Springer; 2002.

[27] Wickham H. Reshaping data with the reshape package. Journal of statistical software. 2007;21(12).

[28] Kolesnikov N, Hastings E, Keays M, Melnichuk O, Tang YA, Williams E, et al ArrayExpress update—simplifying data submissions. Nucleic acids research. 2015;43(Database issue):D1113–6. Epub 2014/11/02. 10.1093/nar/gku1057 25361974 PMC4383899

[29] Piccolo SR, Sun Y, Campbell JD, Lenburg ME, Bild AH, Johnson WE. A single-sample microarray normalization method to facilitate personalized-medicine workflows. Genomics. 2012;100(6):337–44. Epub 2012/09/11. 10.1016/j.ygeno.2012.08.003 ; PubMed Central PMCID: PMCPmc3508193. 22959562 PMC3508193

[30] Shaykhiev R, Wang R, Zwick RK, Hackett NR, Leung R, Moore MAS, et al Airway basal cells of healthy smokers express an embryonic stem cell signature relevant to lung cancer. Stem Cells. 2013;31(9):1992–2002. 10.1002/stem.1459 . 23857717 PMC4051142

[31] Trojani A, Di Camillo B, Tedeschi A, Lodola M, Montesano S, Ricci F, et al Gene expression profiling identifies ARSD as a new marker of disease progression and the sphingolipid metabolism as a potential novel metabolism in chronic lymphocytic leukemia. Cancer biomarkers: section A of Disease markers. 2011;11(1):15–28. Epub 2011/01/01. 10.3233/CBM-2012-0259 . 22820137 PMC13016223

[32] Chen DT, Hernandez JM, Shibata D, McCarthy SM, Humphries LA, Clark W, et al Complementary strand microRNAs mediate acquisition of metastatic potential in colonic adenocarcinoma. Journal of gastrointestinal surgery: official journal of the Society for Surgery of the Alimentary Tract. 2012;16(5):905–12; discussion 12–3. Epub 2012/03/01. 10.1007/s11605-011-1815-0 . 22362069 PMC6753785

[33] Kirzin S, Marisa L, Guimbaud R, De Reynies A, Legrain M, Laurent-Puig P, et al Sporadic early-onset colorectal cancer is a specific sub-type of cancer: a morphological, molecular and genetics study. PLoS One. 2014;9(8):e103159 Epub 2014/08/02. 10.1371/journal.pone.0103159 25083765 PMC4118858

[34] Lambert SR, Mladkova N, Gulati A, Hamoudi R, Purdie K, Cerio R, et al Key differences identified between actinic keratosis and cutaneous squamous cell carcinoma by transcriptome profiling. British journal of cancer. 2014;110(2):520–9. 10.1038/bjc.2013.760 . 24335922 PMC3899778

[35] Wen J, Yang H, Liu MZ, Luo KJ, Liu H, Hu Y, et al Gene expression analysis of pretreatment biopsies predicts the pathological response of esophageal squamous cell carcinomas to neo-chemoradiotherapy. Annals of oncology: official journal of the European Society for Medical Oncology. 2014;25(9):1769–74. Epub 2014/06/08. 10.1093/annonc/mdu201 . 24907633

[36] Gunther OP, Shin H, Ng RT, McMaster WR, McManus BM, Keown PA, et al Novel multivariate methods for integration of genomics and proteomics data: applications in a kidney transplant rejection study. Omics: a journal of integrative biology. 2014;18(11):682–95. Epub 2014/11/12. 10.1089/omi.2014.0062 25387159 PMC4229708

[37] Huffman KM, Koves TR, Hubal MJ, Abouassi H, Beri N, Bateman LA, et al Metabolite signatures of exercise training in human skeletal muscle relate to mitochondrial remodelling and cardiometabolic fitness. Diabetologia. 2014;57(11):2282–95. Epub 2014/08/06. 10.1007/s00125-014-3343-4 25091629 PMC4182127

[38] Berchtold NC, Coleman PD, Cribbs DH, Rogers J, Gillen DL, Cotman CW. Synaptic genes are extensively downregulated across multiple brain regions in normal human aging and Alzheimer's disease. Neurobiology of aging. 2013;34(6):1653–61. Epub 2013/01/01. 10.1016/j.neurobiolaging.2012.11.024 23273601 PMC4022280

[39] Blair LJ, Nordhues BA, Hill SE, Scaglione KM, O'Leary JC 3rd, Fontaine SN, et al Accelerated neurodegeneration through chaperone-mediated oligomerization of tau. The Journal of clinical investigation. 2013;123(10):4158–69. Epub 2013/09/04. 10.1172/JCI69003 23999428 PMC3784538

[40] Sun Y, Caplazi P, Zhang J, Mazloom A, Kummerfeld S, Quinones G, et al PILRalpha negatively regulates mouse inflammatory arthritis. Journal of immunology (Baltimore, Md: 1950). 2014;193(2):860–70. Epub 2014/06/18. 10.4049/jimmunol.1400045 . 24935926

[41] Kool M, Jones DT, Jager N, Northcott PA, Pugh TJ, Hovestadt V, et al Genome sequencing of SHH medulloblastoma predicts genotype-related response to smoothened inhibition. Cancer cell. 2014;25(3):393–405. Epub 2014/03/22. 10.1016/j.ccr.2014.02.004 24651015 PMC4493053

[42] Poschl J, Stark S, Neumann P, Grobner S, Kawauchi D, Jones DT, et al Genomic and transcriptomic analyses match medulloblastoma mouse models to their human counterparts. Acta neuropathologica. 2014;128(1):123–36. Epub 2014/05/30. 10.1007/s00401-014-1297-8 . 24871706

[43] Zhang L, Chen LH, Wan H, Yang R, Wang Z, Feng J, et al Exome sequencing identifies somatic gain-of-function PPM1D mutations in brainstem gliomas. Nat Genet. 2014;46(7):726–30. Epub 2014/06/02. 10.1038/ng.2995 24880341 PMC4073211

[44] Wegert J, Ishaque N, Vardapour R, Georg C, Gu Z, Bieg M, et al Mutations in the SIX1/2 pathway and the DROSHA/DGCR8 miRNA microprocessor complex underlie high-risk blastemal type Wilms tumors. Cancer cell. 2015;27(2):298–311. Epub 2015/02/12. 10.1016/j.ccell.2015.01.002 . 25670083

[45] Lu T, Aron L, Zullo J, Pan Y, Kim H, Chen Y, et al REST and stress resistance in ageing and Alzheimer's disease. Nature. 2014;507(7493):448–54. Epub 2014/03/29. 10.1038/nature13163 24670762 PMC4110979

[46] Tsay JC, Li Z, Yie TA, Wu F, Segal L, Greenberg AK, et al Molecular characterization of the peripheral airway field of cancerization in lung adenocarcinoma. PLoS One. 2015;10(2):e0118132 Epub 2015/02/24. 10.1371/journal.pone.0118132 25705890 PMC4338284

[47] Singh D, Fox SM, Tal-Singer R, Bates S, Riley JH, Celli B. Altered gene expression in blood and sputum in COPD frequent exacerbators in the ECLIPSE cohort. PLoS One. 2014;9(9):e107381 Epub 2014/09/30. 10.1371/journal.pone.0107381 25265030 PMC4179270

[48] Salas S, Brulard C, Terrier P, Ranchere-Vince D, Neuville A, Guillou L, et al Gene Expression Profiling of Desmoid Tumors by cDNA Microarrays and Correlation with Progression-Free Survival. Clinical cancer research: an official journal of the American Association for Cancer Research. 2015;21(18):4194–200. Epub 2015/04/17. 10.1158/1078-0432.ccr-14-2910 . 25878329

[49] Hou J, van Oord G, Groothuismink ZM, Claassen MA, Kreefft K, Zaaraoui-Boutahar F, et al Gene expression profiling to predict and assess the consequences of therapy-induced virus eradication in chronic hepatitis C virus infection. Journal of virology. 2014;88(21):12254–64. Epub 2014/08/08. 10.1128/JVI.00775-14 25100847 PMC4248905

[50] Dhingra N, Shemer A, Correa da Rosa J, Rozenblit M, Fuentes-Duculan J, Gittler JK, et al Molecular profiling of contact dermatitis skin identifies allergen-dependent differences in immune response. The Journal of allergy and clinical immunology. 2014;134(2):362–72. Epub 2014/04/29. 10.1016/j.jaci.2014.03.009 . 24768652

[51] Metzelder SK, Michel C, von Bonin M, Rehberger M, Hessmann E, Inselmann S, et al NFATc1 as a therapeutic target in FLT3-ITD-positive AML. Leukemia. 2015;29(7):1470–7. Epub 2015/05/16. 10.1038/leu.2015.95 . 25976987

[52] Higuchi Y, Kojima M, Ishii G, Aoyagi K, Sasaki H, Ochiai A. Gastrointestinal Fibroblasts Have Specialized, Diverse Transcriptional Phenotypes: A Comprehensive Gene Expression Analysis of Human Fibroblasts. PLoS One. 2015;10(6):e0129241 Epub 2015/06/06. 10.1371/journal.pone.0129241 26046848 PMC4457624

[53] Pajtler KW, Witt H, Sill M, Jones DT, Hovestadt V, Kratochwil F, et al Molecular Classification of Ependymal Tumors across All CNS Compartments, Histopathological Grades, and Age Groups. Cancer cell. 2015;27(5):728–43. Epub 2015/05/13. 10.1016/j.ccell.2015.04.002 25965575 PMC4712639

[54] Wang L, Shen X, Wang Z, Xiao X, Wei P, Wang Q, et al A molecular signature for the prediction of recurrence in colorectal cancer. Molecular cancer. 2015;14:22 Epub 2015/02/04. 10.1186/s12943-015-0296-2 25645394 PMC4320628

[55] Ho DM, Shih CC, Liang ML, Tsai CY, Hsieh TH, Tsai CH, et al Integrated genomics has identified a new AT/RT-like yet INI1-positive brain tumor subtype among primary pediatric embryonal tumors. BMC medical genomics. 2015;8:32 Epub 2015/06/26. 10.1186/s12920-015-0103-3 26109171 PMC4480900

[56] Kang H, Wilson CS, Harvey RC, Chen IM, Murphy MH, Atlas SR, et al Gene expression profiles predictive of outcome and age in infant acute lymphoblastic leukemia: a Children's Oncology Group study. Blood. 2012;119(8):1872–81. Epub 2012/01/03. 10.1182/blood-2011-10-382861 22210879 PMC3293641

[57] Phipson B, Lee S, Majewski IJ, Alexander WS, Smyth GK. ROBUST HYPERPARAMETER ESTIMATION PROTECTS AGAINST HYPERVARIABLE GENES AND IMPROVES POWER TO DETECT DIFFERENTIAL EXPRESSION. The annals of applied statistics. 2016;10(2):946–63. Epub 2017/04/04. 10.1214/16-AOAS920 28367255 PMC5373812

[58] Ritchie ME, Phipson B, Wu D, Hu Y, Law CW, Shi W, et al limma powers differential expression analyses for RNA-sequencing and microarray studies. Nucleic acids research. 2015;43(7):e47 Epub 2015/01/22. 10.1093/nar/gkv007 ; PubMed Central PMCID: PMCPmc4402510. 25605792 PMC4402510

